# Valence-Dependent Belief Updating: Computational Validation

**DOI:** 10.3389/fpsyg.2017.01087

**Published:** 2017-06-29

**Authors:** Bojana Kuzmanovic, Lionel Rigoux

**Affiliations:** ^1^Translational Neurocircuitry Group, Max Planck Institute for Metabolism ResearchCologne, Germany; ^2^Translational Neuromodeling Unit, Institute for Biomedical Engineering, University of Zurich and ETH ZurichZurich, Switzerland

**Keywords:** optimism bias, belief updating, computational modeling, Bayesian theorem, risk judgments, desirability, motivation, probability

## Abstract

People tend to update beliefs about their future outcomes in a valence-dependent way: they are likely to incorporate good news and to neglect bad news. However, belief formation is a complex process which depends not only on motivational factors such as the desire for favorable conclusions, but also on multiple cognitive variables such as prior beliefs, knowledge about personal vulnerabilities and resources, and the size of the probabilities and estimation errors. Thus, we applied computational modeling in order to test for valence-induced biases in updating while formally controlling for relevant cognitive factors. We compared biased and unbiased Bayesian models of belief updating, and specified alternative models based on reinforcement learning. The experiment consisted of 80 trials with 80 different adverse future life events. In each trial, participants estimated the base rate of one of these events and estimated their own risk of experiencing the event before and after being confronted with the actual base rate. Belief updates corresponded to the difference between the two self-risk estimates. Valence-dependent updating was assessed by comparing trials with good news (better-than-expected base rates) with trials with bad news (worse-than-expected base rates). After receiving bad relative to good news, participants' updates were smaller and deviated more strongly from rational Bayesian predictions, indicating a valence-induced bias. Model comparison revealed that the biased (i.e., optimistic) Bayesian model of belief updating better accounted for data than the unbiased (i.e., rational) Bayesian model, confirming that the valence of the new information influenced the amount of updating. Moreover, alternative computational modeling based on reinforcement learning demonstrated higher learning rates for good than for bad news, as well as a moderating role of personal knowledge. Finally, in this specific experimental context, the approach based on reinforcement learning was superior to the Bayesian approach. The computational validation of valence-dependent belief updating represents a novel support for a genuine optimism bias in human belief formation. Moreover, the precise control of relevant cognitive variables justifies the conclusion that the motivation to adopt the most favorable self-referential conclusions biases human judgments.

## Introduction

A growing body of research has demonstrated that people update their beliefs about future outcomes in an asymmetric manner: they tend to neglect undesirable information, but take desirable information more readily into account (Eil and Rao, 2011; Sharot et al., 2011; Korn et al., 2012; Garrett and Sharot, 2014; Kuzmanovic et al., 2015, 2016a,b; Sharot and Garrett, 2016a). Similar asymmetric belief updating has been shown in the context of reinforcement learning. When learning about reward probabilities of different choice options (represented by neutral stimuli), people tended to learn more from better-than-expected outcomes (positive prediction errors) than from worse-than-expected outcomes (negative prediction error) (Palminteri et al., 2016; Lefebvre et al., 2017)^1^.

As proposed by the differential scrutiny account, people tend to accept easily information with favorable implications (“*Can* I believe this?”), but are likely to apply an effortful scrutiny when considering unfavorable information (“*Must* I believe this?”) (Gilovich, 1991; Krizan and Windschitl, 2007; Shepperd et al., 2008). Such standards of proof need not be voluntary, as the good news-bad news effect does not rely on a conscious report. Rather, it is quantified by exploiting actual belief updating behavior at the individual level. Moreover, even those individuals who demonstrate biased belief updating were not aware of it (Kuzmanovic et al., 2015, 2016a). However, in contrast to reinforcement learning studies, where belief updating is informed solely by the history of previous choices, predictions about future life events are determined by multiple and interrelated factors. Thus, differential information evaluation could result from the motivation to draw desirable conclusions and to adopt the most favorable future outlook (Kunda, 1990; Shepperd et al., 2002; Sharot et al., 2011). Alternatively, it could also be explained by cognitive factors relating to various aspects of the available information such as the size of expected and presented probabilities, as well as prior knowledge about hazards under consideration, and about personal vulnerabilities and resources (Krizan and Windschitl, 2007; Shah et al., 2016).

In the context of such complex processing, modeling acts as a computational microscope and provides an efficient method for isolating influential factors with maximal precision. Formalizing competing models with varying components of belief updating allows for the exact specification of hypotheses about possible mechanistic causes of the observed behavior, and for the identification of those components that substantially influence update dynamics. Moreover, the possibility of controlling for relevant cognitive variables on a trial-by-trial basis increases the confidence in conclusions about motivational explanations for asymmetric updating.

Hence, in order to provide a formal proof that belief updates are biased by the valence of new information, the present study combines an established belief update paradigm with computational modeling. We assessed beliefs about average and personal risks of negative future life events (e.g., cancer or car theft) and their updates in response to good news (e.g., a lower base rate of cancer than expected), or bad news (e.g., a higher base rate of cancer than expected). First, we applied previous formalizations of *unbiased* Bayesian belief updating (according to Shah et al., 2016) to compare actual participants' behavior to a normative benchmark. Second, we specified a competitive Bayesian model that allows for *biased* belief updating to investigate whether actual updates are better explained by a biased or by an unbiased model. And third, we formalized an alternative computational model of belief updating that relied on reinforcement learning. The selection of the alternative model that best fits the data allowed us to test formally whether learning rates were higher in response to good news than to bad news, and whether participants' personal knowledge (how they perceive their risk relative to the average risk) systematically affected the consideration of the new information (actual base rates). Together, these analyses validated the optimism bias in human belief formation. Participants' belief updates were asymmetric, and deviated more from the normative Bayesian benchmark in response to bad news relative to good news. Moreover, comparison of the different computational models confirmed the valence-induced bias in belief updating, and the need to include personal knowledge in update dynamics.

## Materials and methods

### Participants

The Exploratory Software for Confidence Intervals (Cumming, 2014) indicated a sample size of *N* = 27 to be required to achieve an average target margin of error in standard deviation units of 0.4, with the correlation between repeated measures of 0.5, based on prior work on optimism bias in belief updating, that also assessed participants' beliefs about base rates (but in a separate experimental session and not within one trial, Kuzmanovic et al., 2015, Study 2). Thirty participants were recruited from the Max Planck Institute for Metabolism Research subject pool. One participant was excluded because she recognized that the base rates were manipulated (see Section Procedure), one because he made no updates in 84% of trials, and one because she made updates away from the presented BR in 18% of trials indicating problems with understanding the task (for instance, if eBR = 10% and E1 = 10% and the presented BR is 5%, the update away from the BR would be 15%). Consequently, 27 participants were included in the analysis (age *M* = 28.67, *SD* = 5.17, 15 males). All procedures were in accordance with the World Medical Association Declaration of Helsinki and were approved by the local ethics committee of the Medical Faculty of the University of Cologne, Germany. All subjects gave written informed consent.

### Procedure

The experiment was conducted during an acquisition of fMRI scans (neuroimaging data not reported here) using Presentation 18.1 (Neurobehavioral Systems), and consisted of 80 trials with 80 different adverse life events (e.g., cancer or car theft; for a complete list see Supplementary Material). Before the experiment, all participants received written instructions, and completed six practice trials with stimulus events not used in the experiment.

**Figure 1:**
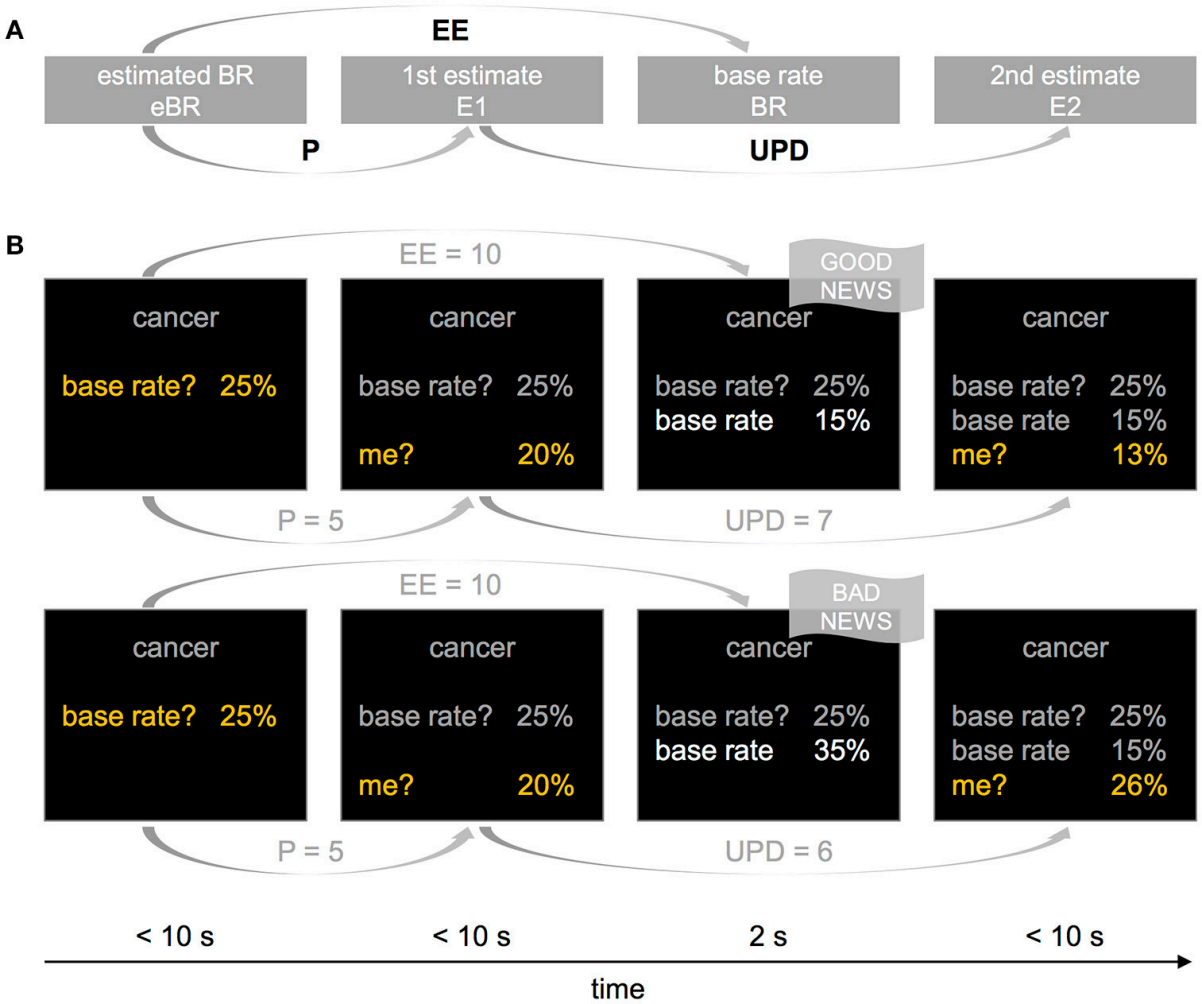
The general structure of the experimental design **(A)** and example trials with good and bad news **(B)**. **(A)** In each trial and with respect to each of the 80 stimulus events, participants (i) estimated the base rate (eBR), (ii) estimated their lifetime risk (E1), (iii) were provided with the “actual” base rate (BR), and (iv) re-estimated their lifetime risk (E2). Estimation errors (EE) were computed as the absolute difference between eBR and BR, and updates (UPD) as the expected shift from E1 to E2 (i.e., E1–E2 when BR < eBR, and E2–E1 when BR > eBR). The difference between eBR and E1 indicates how much the participant believes that he or she deviates from the average (personal knowledge, P). **(B)** In the upper row, a participant is presented with a lower base rate than expected (BR < eBR), providing her with good news. In the upper row, the presented base rate is higher than expected, indicating bad news.

The task relied on the belief update methodology used in previous studies (see Figure 1; e.g., Sharot et al., 2011; Kuzmanovic et al., 2016a). Participants began each trial with estimating the base rate (eBR) of a negative life event (i.e., the average probability of the respective event happening to an individual living in the same socio-cultural environment). Next, they were asked to estimate their own likelihood of experiencing the life event in their lifetime (first estimate, E1), and were subsequently presented with the actual base rate (BR). At the end of each trial, participants had to re-estimate their own risk (second estimate, E2).

The difference between E1 and E2 corresponds to the size of the update. The difference between eBR and BR indicates the size of the estimation error (EE), and whether BR was favorable (better than expected), or unfavorable (worse than expected). Moreover, the difference between eBR and E1 allows to infer how similar a participant perceives herself relative to the average person (e.g., E1 < eBR indicates that a person believes that she is less at risk than the average person, and vice versa). This may be important as base rates may become irrelevant if one assumes oneself to be very different from the average person (e.g., somebody without a car will not be concerned about the average risk of car theft, see Shah et al., 2016). Such personal knowledge can be protective (e.g., having a healthy lifestyle), or can make a person more vulnerable to a hazard (e.g., having a family history of cancer). Further methodological implications related to eBR are described in detail elsewhere (see Garrett and Sharot, 2014; Kuzmanovic et al., 2015; Shah et al., 2016; Sharot and Garrett, 2016b). Hence, we included personal knowledge into the formalization of the alternative computational models (see Section Computational Modeling).

All events (eBR, E1, BR, E2) were incorporated into one trial, and the full history of outcomes was displayed at all times, in order to avoid confounds by memory load and errors. Were these estimates assessed in separate sessions, participants would not be able to remember their exact previous estimates and the presented base rates for all 80 stimulus events. Such memory errors have been reported to be equal for trials with good and bad news (Sharot et al., 2011; Garrett and Sharot, 2014), so that they do not represent a confound for the valence-induced bias in updating. However, in the context of computational modeling, they would increase the noise in the data and thus impede the computational inference of the most likely parameter values causing the observed updates, and should therefore be avoided.

Unbeknownst to participants, we manipulated BR in order to be able to control the number of trials and the size of EE across conditions (see Table 1 for mean EE, and for more details about the algorithm used to generate manipulated EE, see Supplementary Material). Moreover, this allowed us to randomly assign the 80 stimulus events to different valences and error sizes, anew for each subject. In this way, cognitive effects such as prior experiences with the future events or their personal meanings were likely to be balanced across conditions (e.g., see Kuzmanovic et al., 2015 for tests of equal distributions). BRs were capped between 1 and 90% (because base rates greater than 90% would not be credible for the majority of stimulus events), and were introduced as the probability of the respective event occurring to persons of the same sex and age, living in the same socio-cultural environment as the participant. In a final debriefing after the experiment, a funneled procedure was used to ensure that subjects did not suspect the manipulation of the base rates, or the purpose of the study.

**Table 1:** Task and model parameters, their statistics and sources.

**Parameter**	***M* (*SD*)**		***p***	**Source**
	**Good news**	**Bad news**		
Number of trials	39.37 (2.24)	37.96 (1.85)	0.015	
Estimated base rate (eBR)	47.90 (11.57)	44.81 (10.82)	0.002	*Participant's response*
First estimate (E1)	41.77 (12.38)	37.85 (10.83)	0.004	*Participant's response*
Personal knowledge (P)	6.12 (6.13)	6.96 (7.81)	0.282	P = eBR - E1
Presented base rate (BR)	34.44 (11.24)	58.69 (10.85)	0.000	*Base rate algorithm*^†^
Estimation error (EE)	13.45 (0.91)	13.86 (0.65)	0.006	EE = |eBR - BR|
Second estimate (E2)	34.24 (11.55)	44.30 (12.21)	0.000	*Participant's response*
RT E2 in sec	3.19 (1.04)	3.17 (1.06)	0.960	*Participant's response*
Actual update (UPD)	7.53 (2.66)	6.45 (2.49)	0.024	UPD_GOOD_ = E1 - E2,
				UPD_BAD_ = E2 - E1
Likelihood ratio (LHR)	1.56 (1.19)	1.54 (2.32)	0.964	$LHR=\;\frac{E1\;}{1-E1}÷\frac{eBR\;}{1-eBR}$
% of trials with LHR < 1	55.92 (19.29)	56.00 (19.98)	0.976	
Bayesian E2 (E2b)	30.74 (10.86)	49.82 (12.15)	0.000	$\text{E}2\text{b }=\;\frac{\text{BR }*\text{ LHR}}{\text{BR }*\text{ LHR }+\left(1\;-\text{ BR}\right)}$
Bayesian UPD (UPDb)	11.04 (1.95)	11.97 (1.90)	0.003	UPDb_GOOD_ = E1 - E2b,
				UPDb_BAD_ = Eb2 - E1
‘Optimistic’ UPDb (UPDbo)	7.60 (2.47)	6.48 (2.38)	0.006	UPDbo_GOOD_ = UPDb * (S + A),
				UPDbo_BAD_ = UPDb * (S – A)

*All measures (except for number of trials) were provided by participants or computed at each trial, and were then averaged separately for the conditions GOOD and BAD, separately for each participant. Positive UPD values indicated updates toward the BR, and negative values updates away from the BR (<2% of the trials). P: scores > 0 indicate lower risk than the average person, 0 indicates an equal risk, scores <0 indicate greater risk. LHR, likelihood ratio; LHR scores <1 indicate lower risk than the average person, 1 indicates an equal risk, scores > 1 indicate greater risk. LHR, E2b and UPDb: according to Shah et al. (2016). S and A, free model parameters Scaling (μ = 1) and Asymmetry (μ = 0). p values refer to paired two-tailed t-tests with n = 27*.

† *see Supplementary Material*.

Participants were free to report a probability anywhere between 1 and 99%. Starting from 50% in eBR, they selected the desired probability within this range by using two buttons to increase or decrease the number displayed on the screen (Figure 1B, yellow font color), and a third button to affirm finally the selected choice. The final confirmation of the selected number with a button press aimed to reduce response noise. In E1, the starting number equaled the one selected in eBR, and in E2, the starting number corresponded to the one selected in E1.

### Analysis of task performance

Statistics for all task-related parameters are listed in Table 1. In each trial, update (UPD) was computed as the difference between E1 and E2 such that positive values indicated an update in the direction suggested by the new information (i.e., lower BR than expected suggested lower E2 than E1 and vice versa; see Table 1). Trials were divided into those in which participants received “good news” (GOOD; BR < eBR, BR lower than expected), and those in which participants received “bad news” (BAD; BR > eBR, BR higher than expected). Given that in a normative sense estimation errors *generally* signal that related beliefs need to be updated (independently of the valence of the error), biased updating was assessed by comparing updates in GOOD-trials with those made in BAD-trials. Furthermore, for each participant, we conducted a linear regression to predict his or her updates on each trial using valence of news (GOOD vs. BAD), while including eBR, E1, and EE as covariates (all measures z-scored within subject; note that the additional inclusion of BR is obsolete as it is a linear combination of E1 and EE). Finally, in addition to extracting participants' actual updates, we simulated rational Bayesian updates—according to Shah et al. (2016), to establish a normative benchmark for belief updating (“Bayesian updates,” UPDb, see Table 1).

When comparing repeated measures throughout the study, we used paired *t*-tests (two-tailed), and the standard deviation of the paired differences as a standardizer for Cohen's *d* (Cumming, 2014). Following trials were excluded prior to all analysis: (i) trials with a missing response (on average 1.3 trials per subject), (ii) trials with EE = 0 (on average 1.0 trial per subject; e.g., in a GOOD trial, when eBR was 1%, no lower BR than 1% could be presented), and (iii) trials with updates deviating more than 4 *SD* from subject's mean update (2 excluded trials in total).

### Computational modeling

We implemented competing computational cognitive models, and assessed which model provided the best account for participants' actual data. More precisely, each model was first fitted to the data using Bayesian variational inference. This procedure yields for each hypothesis (a) a posterior distribution across the parameters, and (b) an approximation to the evidence of the model. The posterior provides sufficient statistics (i.e., mean and variance) of the parameter estimates. The model evidence reflects the goodness of fit of the model, which is penalized for the complexity of the parametrization. Here, we use the Free-energy approximation that has been shown to be superior to other approximations like AIC or BIC (Penny, 2012). For each analysis, (approximate) model evidences of all subjects and all tested models were then entered in a random-effect Bayesian model comparison procedure. This scheme allows us to infer the probability of each subject to be best described by the respective models (model attributions), and therefore to estimate the frequency of each model in the population (estimated model frequency, *Ef*). From there, we computed the protected exceedance probability (*pxp*) of each model, which is the probability that the hypothesis predominates in the population, above and beyond chance (see Rigoux et al., 2014 for more details). Notably, the model-based approach allows us to control formally for potential differences in trial-wise eBR, E1, and EE across conditions. The Bayesian model comparison was performed using the VBA toolbox (Daunizeau et al., 2014).

#### “Rational” and “optimistic” Bayesian models of belief updating

We compared models formalizing “rational” (according to Shah et al., 2016) and “optimistic” (i.e., valence-dependent) Bayesian updating. Starting from the “rational” Bayesian model (see Table 1, UPDb), we included two free parameters, Scaling and Asymmetry (S and A; see Table 1, UPDbo), that were estimated separately for each participant dependent on their trial-by-trial behavior. Scaling indicates the average tendency of a participant to update initial beliefs due to the presented base rate relative to what is predicted by Bayes' rule (S <1 leads to lower updating, and S > 1 to greater updating). Asymmetry renders Scaling differentially for bad and good news. More precisely, A > 0 leads to larger updates for good than for bad news, representing an optimism bias, again relative to the rational Bayesian prediction. Thus, setting the prior values strictly to 1 for Scaling and 0 for Asymmetry specifies the null hypothesis that update is equal to the predictions of the “rational” Bayesian model. The alternative hypothesis is that these parameters are different from the null hypothesis and have an influence on the update, and thus need to be estimated for each participant (i.e., included into the model as free parameters). We formalized four models that resulted from the possible parameter combinations (S+A, S, A, Ø; S and A indicate that the respective parameter was estimated instead of being fixed), and applied Bayesian model comparison (Rigoux et al., 2014) to assess which of these models best accounted for participants' behavior.

#### Alternative computational models of belief updating

Furthermore, we formalized an alternative, more simple computational model of belief updating. It relied on the generic form of reinforcement learning (Sutton and Bart, 1998) with remarkably simple interpretability: belief update is proportional to the prediction error (weighted by the learning rate Alpha, indicating the general tendency of each participant to take prediction errors into account). In the context of belief updating, EE can be considered as equivalent to the prediction error in classical reinforcement learning. To account for valence-dependent asymmetry in belief updating, the learning rate Alpha was estimated separately for good and bad news (Palminteri et al., 2016; Lefebvre et al., 2017). In addition, we took personal knowledge into account by weighting EE by the relative personal knowledge (rP). Thus, the alternative computational model (*m1*) takes the following form:

$$\begin{array}{lll} UPD & = & LR^{*}EE^{*}\left(1-rP^{*}W\right)\\ LR_{GOOD} & = & Alpha+Asymmetry\\ LR_{BAD} & = & Alpha-Asymmetry \end{array}$$

Learning rate (LR, general tendency of each participant to update her beliefs in response to EE) smaller than 1 indicates updates smaller than EE, and LR greater than 1 indicates updates greater than EE (while EE is also weighted by a function of rP, see below). Furthermore, the learning rate was formalized as a function of the valence of news. That is, it was expected to differ systematically for GOOD and BAD (i.e., lower learning rates and smaller updates for BAD than for GOOD). If Asymmetry is different from zero, then there is an effect of Valence, and the influence of EE is different for GOOD and BAD.

RP stands for *relative* personal knowledge, i.e., the difference between eBR and E1 relative to the maximal possible difference in each trial. It weights the impact of EE, because the more a person thinks that she deviates from the average, the less influential the presented BR should be for her judgment. Weight (W) influences rP to allow for a formal testing of the potential effect of rP on belief updating. If Weight is different from 0, then rP has an effect on belief updating. RP ranged from 0 to 1, with rP = 0 when eBR = E1, and rP = 1 when |eBR – E1| is maximal. For instance, when eBR = 15%, E1 of 1% generates the maximal possible difference *lower* than eBR of 14%, and E1 of 99% generates the maximal possible difference *higher* than eBR of 84%, given that participants' estimates were capped between 1 and 99%. Therefore, for each trial, rP was computed as follows:

$$\begin{array}{lll} if\;E1<eBR:rP & = & \left(eBR-E1\right)/\left(eBR-1\right)\\ if\;E1>eBR:rP & = & \left(E1-eBR\right)/\left(99-eBR\right)\\ if\;E1=eBR:rP & = & 0 \end{array}$$

In order to test the respective effects of learning rate, valence, and personal knowledge, we generated all possible variations of the update equation by switching on (by letting the parameter free), or off (by fixing the parameter's prior variance to zero) the parameters Alpha, Asymmetry, and Weight. Note that by setting Asymmetry and Weight to 0 we obtain the classical reinforcement learning rule (UPD = Alpha ^*^ EE), and prior expectations of 0 for Asymmetry and Weight and 1 for Alpha specify the null hypothesis UPD = EE. In the alternative hypothesis, those parameters have an influence on update, and thus need to be estimated for each participant (i.e., included into the model as free parameters).

Eight models with all possible parameter combinations (α+A+W, α+A, α+W, α, A+W, A, W, Ø; α, A and W indicate that the respective parameter was estimated instead of being fixed) were estimated for each subject and were then entered in a random effect Bayesian model comparison procedure. Finally, we compared the winning alternative model to the winning Bayesian model.

## Results

### Comparison of actual updates and Bayesian updates

**Figure 2:**
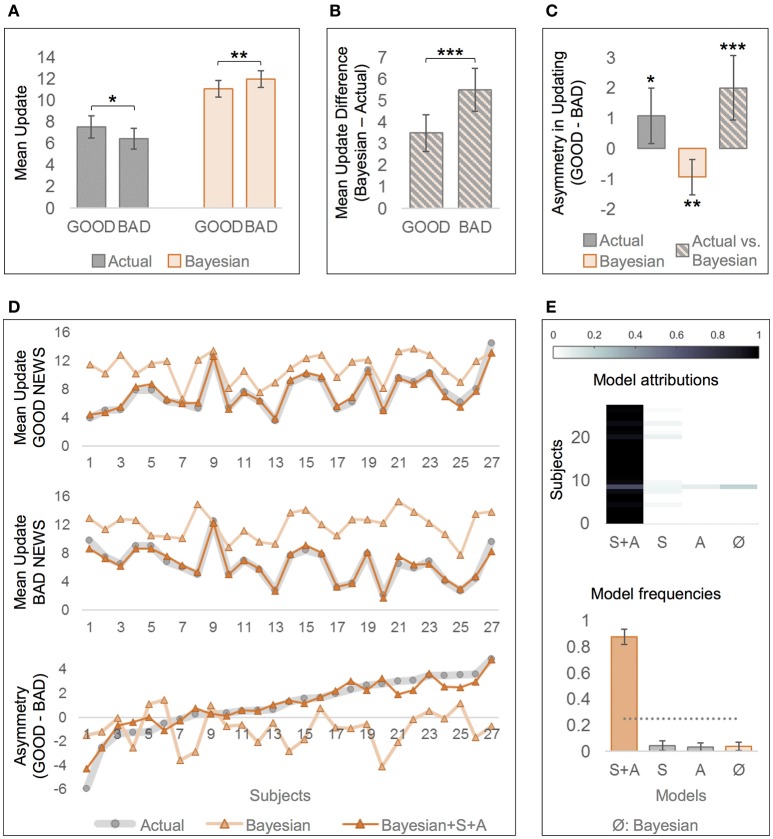
Comparison of actual and Bayesian updates, and of “rational” and “optimistic” Bayesian models of belief updating. **(A)** Actual updates were larger after good news (GOOD) than after bad news (BAD), indicating an optimism bias, but Bayesian updates were larger after bad than after good news. **(B)** The difference between the Bayesian and the actual update was greater for BAD than for GOOD trials. Note that Bayesian updates were generally higher than actual updates. **(C)** Measures of asymmetric updating (mean update in GOOD—mean update in BAD), that are derived from **(A,B)**. While there is an optimistic asymmetry in actual updates, and an opposite asymmetry in Bayesian updates, contrasting the asymmetry in actual updates with the one in Bayesian updates reveals a larger optimism bias than when considering the actual updates alone. **(A–C)** Error bars show 95% CI. **(D)** Differences in mean updates and asymmetry in updating between the actual data and the predictions by the two computational models: “rational” Bayesian (according to Shah et al., 2016), and “optimistic” Bayesian including the free parameters Asymmetry **(A)** and Scaling (S, Bayesian+A+S). Subjects are sorted by asymmetry in updating based on actual data (bottom, gray line) in ascending order. **(E)** “Optimistic” Bayesian model (“A+S”) accounts better for actual data than the “rational” Bayesian model (“Ø,” according to Shah et al., 2016), or other less complex models (“S” and “A”). Labels at the x-axis indicate which parameters are left free. Posterior model attribution (top): Each colored cell gives the posterior probability that a given subject (y-axis) is best explained by a specific model (x-axis). The more contrasted a line, the better the confidence in the attribution. Posterior model frequencies (bottom): Each bar represents the expected frequency of a model in the tested sample, i.e., how many subjects are expected to be best described by a model (error bars show standard deviation). The gray dashed line represents the null hypothesis, namely that all models are equally likely in the population (chance level). ^*^p < 0.05, ^**^p < 0.01, ^***^p < 0.001.

With respect to actual behavior, bad news led to smaller updates than good news indicating an optimism bias, *t*_(26)_ = 2.42, *p* = 0.024, *d* = 0.48, *M* = 1.08, *SD* = 2.31, 95% CI [0.16, 1.99], see Figure 2A for mean updates and Figure 2C for the respective difference measures. By contrast, simulated Bayesian updates showed the opposite asymmetry in updating, *t*_(26)_ = −3.28, *p* = 0.003, *d* = 0.63, *M* = −0.93, *SD* = 1.48, [−1.51, −0.35]. Moreover, while the actual updates generally deviated from Bayesian updates (dUPD = UPDb – UPD), dUPD_GOOD_: *t*_(26)_ = 8.48, *p* < 0.001, *d* = 1.63, *M* = 3.51, *SD* = 2.15, [2.66, 4.36], dUPD_BAD_: *t*_(26)_ = 11.36, *p* < 0.001, *d* = 2.19, *M* = 5.51, *SD* = 2.52, [4.52, 6.51], this deviation was greater in BAD than in GOOD, *t*_(26)_ = 3.91, *p* = 0.001, *d* = 0.76, *M* = 2.01, *SD* = 2.66, [0.95, 3.06], see Figures 2B,C. This indicates that participants' updates deviated more strongly from the Bayes' normative benchmark after bad news than after good news (see also Figure 2D, first two plots, for greater descriptive deviations from Bayesian updates for BAD than for GOOD). The larger deviation from the normative benchmark in BAD than in GOOD trials reflects a relative optimism bias, that contrasts (i) the actual asymmetry in updating with (ii) the asymmetry predicted by the Bayes' theorem, see Figure 2C, “Actual vs. Bayesian.” Figure 2D (bottom) can be examined for examples of participants with similar actual asymmetry in updating but different Bayesian asymmetry, resulting in different relative optimism biases (e.g., subjects 25 and 26).

Linear regression analyses revealed that updates were larger in BAD than in GOOD trials even after controlling for trial-wise EE, *t*_(26)_ = −2.73, *p* = 0.012, *d* = 0.53, *M*_β*valence*_ = −0.18, *SD* = 0.35, 95% CI [−0.32, −0.05], or for eBR, E1 and EE, *t*_(26)_ = −2.49, *p* = 0.020, *d* = 0.48, *M*_β*valence*_ = −0.18, *SD* = 0.38, [−0.34, −0.03], confirming the optimism bias in the observed data.

### Comparison of “rational” and “optimistic” Bayesian models of belief updating

Bayesian model comparison of all four Bayesian models revealed that the “optimistic” Bayesian model that included both Scaling and Asymmetry as free parameters best accounted for the actual participants' behavior (*Ef* = 0.88, *pxp* = 1; see Figures 2D,E). Moreover, *t*-tests showed that Scaling was considerably smaller than 1, *t*_(26)_ = −13.05, *p* < 0.001, *d* = −2.60, mean difference to 1 was −0.39, *SD* = 0.15, 95% CI [−0.45, −0.33], showing that participants indeed updated less than predicted by Bayes' rule. Furthermore, Asymmetry was considerably larger than zero, *t*_(26)_ = 4.12, *p* < 0.001, *d* = 0.78, *M* = 0.07, *SD* = 0.09, [0.03, 0.10], confirming that participants made smaller updates after bad news than after good news.

### Alternative computational models of belief updating

**Figure 3:**
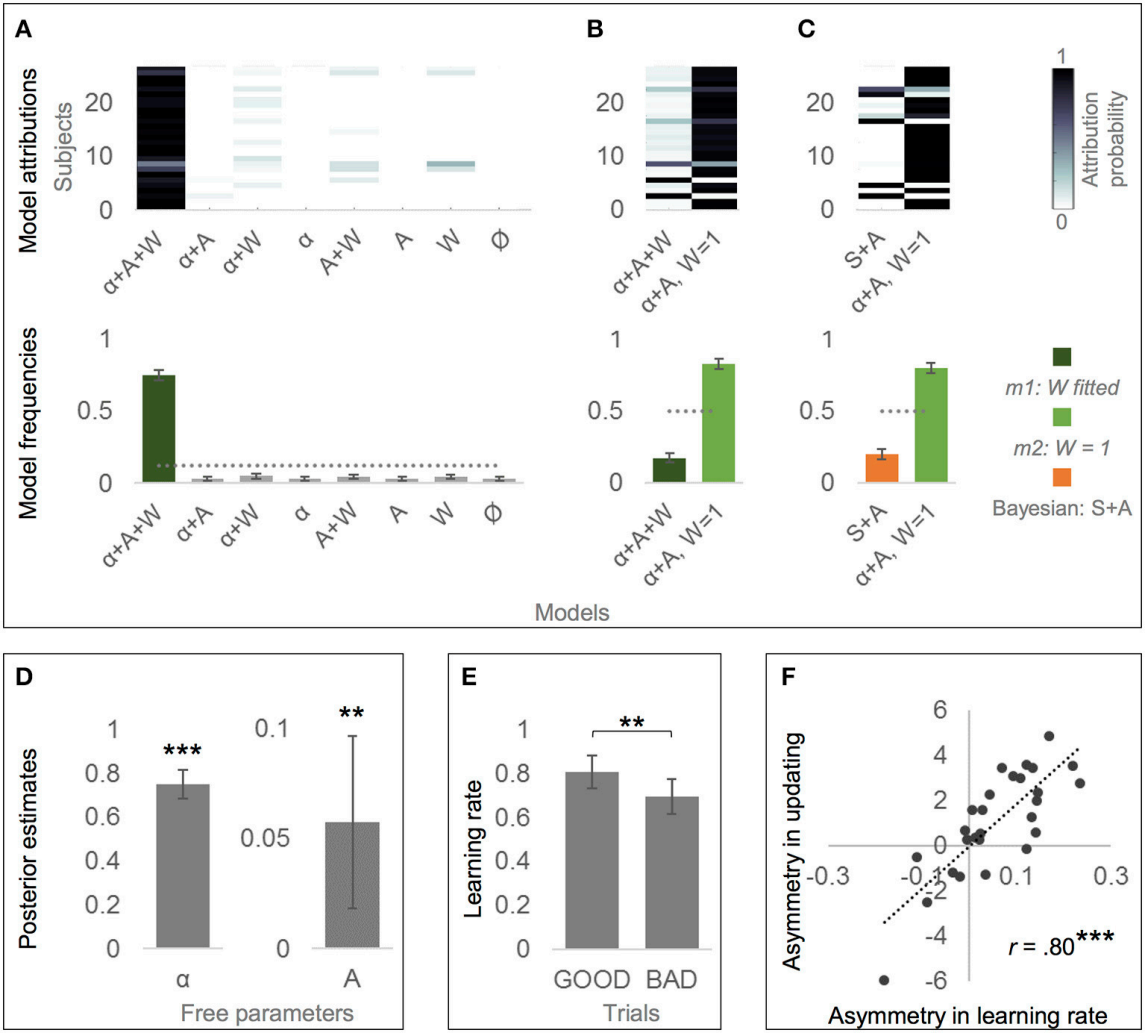
Alternative model of belief updating based on classical reinforcement learning. **(A)** The alternative model that incorporated the effects of learning rate (α ≠ 1), valence (Asymmetry, A ≠ 0) and personal knowledge (W ≠ 0) best accounted for the actual data (“α+A+W,” *m1*). Thus, it provided a formal test that all three factors are influential components in belief updating. Labels α, A and W indicate which parameters are left free. Ø indicates the null hypothesis, namely that there is no effect of learning rate (α = 1), valence (A = 0) or personal knowledge (W = 0), and thus that update is simply proportional to estimation error. **(B)** The simpler version of the alternative model that fixes W to 1 (*m2*; i.e., personal knowledge is influential, but equally across subjects) outperformed *m1* (W formalized as a free parameter with a prior of 0). Thus, *m2* is the finally resulting alternative model of belief updating. **(C)** The winning alternative model (*m2*) accounts better for the actual data than the winning “optimistic” Bayesian model (“Bayesian: S+A”). **(A–C)** Posterior model attribution (top): Each colored cell gives the posterior probability that a given subject (y-axis) is best explained by a specific model (x-axis). Posterior model frequencies (bottom): Each bar represents the expected frequency of a model in the tested sample, i.e., how many subjects are expected to be best described by a model (error bars show standard deviation). The gray dashed line represents the null hypothesis, namely that all models are equally likely in the population (chance level). **(D)** Mean posterior parameter estimates of the learning rate resulting from model *m2*. Alpha (α) was significantly smaller than 1, indicating that updates were lower than the estimation error (weighted by personal knowledge). Asymmetry **(A)** was significantly greater than 0, supporting the effect of valence as learning rates were larger for good than for bad news. **(E)** Learning rates resulting from model *m2* were larger in response to good than to bad news (GOOD and BAD trials), confirming the effect of valence on belief updating. **(D,E)** Error bars show 95% CI. **(F)** Across subjects, estimated Asymmetry in learning rate **(A)** derived from *m2* correlated with the asymmetry in updating derived from the actual data. ^**^p < 0.01, ^**^p < 0.001.

Bayesian model comparison of all eight alternative models revealed that the alternative model that included the effects of learing rate, valence and personal knowledge (*m1*, free parameters α, A, and W) predicted actual belief updates better than all simpler versions of the model, *Ef* = 0.75, *pxp* = 1 (see Figure 3A). Subsequent *t*-tests showed that Alpha was considerably smaller than 1 {*M* = 0.73, *SD* = 0.17, *t*_(26)_ = −8.33, *p* = 0.000, *d* = 4.35, estimated difference to 1 was −0.27, 95% CI [-0.34, −0.20]}, showing that participants were indeed updating less than predicted by EE. Furthermore, Asymmetry was considerably larger than zero {*M* = 0.06, *SD* = 0.10, *t*_(26)_ = 2.99, *p* = 0.006, *d* = 0.57, 95% CI [0.02, 0.09]}, indicating that participants disregarded EE even more in response to bad news than good news. And finally, W was considerably larger than zero {*M* = 0.86, *SD* = 0.25, *t*_(26)_ = 18.11, *p* = 0.000, *d* = 3.48, 95% CI [0.76, 0.95]}, demonstrating that personal knowledge indeed influenced participants' update behavior.

However, because W was relatively close to 1, and because α and W may not be completely orthogonal, we tested whether *m1* (all three parameters free) or *m2* (α and A are free parameters, but W is fixed to 1) provides a better explanation of the data. Indeed, *m2* had a better performance than *m1, Ef* = 0.83, *pxp* = 0.967, see Figure 3B. Comparison of all four possible versions of *m2* (α either free or 1, A either free or 0) also confirmed that *m2* including the effects of learning rate and valence outperformed simpler *m2* versions (*Ef* = 0.78, *pxp* = 1). This shows that the influence of personal knowledge on updating can simply be formalized as *(1 – rP)* instead of *(1 – rP* ^*^ *W)* because W is equal 1. In other words, there is no need to allow for deviations from the *(1 – rP)* rule, or for inter-individual variance in these deviations. As a consequence, the finally resulting model of belief updating (*m2*) takes the following form:

$$\begin{array}{l} UPD=LR^{*}EE^{*}\left(1-rP\right) \end{array}$$

Finally, Bayesian model comparison revealed that *m2* (free parameters α and A, *W* = 1) predicted actual belief updates even better than the “optimistic” Bayesian model (free parameters S and A), *Ef* = 0.80, *pxp* = 0.987, see Figure 3C.

Fitting *m2* to data across subjects again revealed that α was considerably smaller than 1 {*M* = 0.75, *SD* = 0.17, *t*_(26)_ = −7.85, *p* = .000, *d* = −1.47, estimated difference to 1 was −0.25, 95% CI [-0.32, −0.19]}, confirming that participants updated less than predicted by EE, see Figure 3D. Furthermore, Asymmetry was again considerably larger than zero {*M* = 0.06, *SD* = 0.10, *t*_(26)_ = 3.00, *p* = 0.006, *d* = 0.60, 95% CI [0.02, 0.10]; see Figure 3D}, indicating that participants disregarded EE even more in response to bad news than good news (Figure 3D). In consequence, learning rates were lower in BAD (*M* = 0.69, *SD* = 0.20) than in GOOD (*M* = 0.81, *SD* = 0.19) trials, see Figure 3E. In addition, Asymmetry (*r* = 0.80, *p* < 0.001), but not α (*r* = − 0.04, *p* = 0.837) correlated with the optimism bias (asymmetry in updating) across subjects (see Figure 3F).

## Discussion

The present study provides converging evidence for valence-dependent belief updating. Participants updated their beliefs about hazards more in response to desirable new information than in response to undesirable information. The good news-bad news effect was significant even after controlling for prior beliefs and their violations (by including estimation errors and initial estimates of risks and base rates as covariates in a regression analysis). Moreover, participants updates were compared to simulated belief updates expected to be made by a “dispassionate thinker—one who is not swayed by desires for any particular outcome” (Krizan and Windschitl, 2007, p. 107), according to a Bayesian model of belief updating (Shah et al., 2016). Optimism bias was present only in actual updates, but not in simulated updates. Furthermore, in line with previous results (Sharot and Garrett, 2016b), participants showed an increased resistance to rationally expected belief change particularly when they received bad news compared to good news. Thus, the valence-dependent belief updating supports the notion that motivational factors may guide information processing to allow to discredit threatening news (Kunda, 1990).

Our findings differ from what has been reported by Shah et al. (2016) in some instances. First, while our Bayesian update simulation showed larger updates in response to bad than to good news, some of the Bayesian update simulations reported by Shah and colleagues showed the opposite pattern. This is not a true inconsistency because these specific simulations by Shah and colleagues were based on *ad hoc* samples of artificial agents with predefined personal knowledge and estimation error distributions. In contrast, our Bayesian update simulations were informed by actual data recorded during the experiments (presented base rates and likelihood ratios, the latter calculated based on participants' initial self-estimates and their base rate estimates). Importantly, the assumptions by Shah et al. (2016) about the distribution of the personal knowledge have been challenged by our data, which showed that participants assumed to be less at risk than average only for a half of events (56% likelihood ratio <1), see also Sharot and Garrett (2016b) for similar findings. We also showed that the stimulus events were not perceived to be extremely rare (mean estimated average likelihood was 46%). Second, greater deviation of participants' updates from the Bayesian benchmark in response to bad than to good news was not consistently evident in experiments by Shah and colleagues (calculated based on actual experimental data with negative life events, experiments 2–4; see Sharot and Garrett (2016b) for the discussion of positive events)^2^. Such inconsistencies are likely to be caused by methodological factors (e.g., highly variable numbers of events and subjects; memory errors due to task structure with two separate sessions for first and second self-estimates), that reduced the statistical power and yielded nonsignificant results, which is no proof for an absence of effect.

The conclusion that the differential evaluation of available evidence mediates the influence of desires on predictions is in line with recent neuroscientific research (Sharot and Garrett, 2016a). Persons scoring high on trait optimism demonstrated reduced neural tracking of undesirable estimation errors relative to low-optimistic individuals (Sharot et al., 2011). Furthermore, differential processing of desirable and undesirable errors has been related to differences in the structural connectivity of frontal-subcortical circuits linking cognitive and emotional processing (Moutsiana et al., 2015). Motivational explanations are also supported by the finding of an enhanced optimism bias after dopaminergic intervention (Sharot et al., 2012). Moreover, favorable updating indeed seems to have a positive subjective value as it has been associated with an increased activation of the ventromedial prefrontal cortex known to represent reward values (Kuzmanovic et al., 2016a).

The valence-induced asymmetry in updating was additionally validated by the refinement of existing computational models of belief updating under formal control of trial-wise task parameters (i.e., own risk estimate, estimated base rate, actual base rate and the resulting estimation error). First, we demonstrated that an optimistically biased Bayesian model better accounted for the data than the fully rational Bayesian model (according to Shah et al., 2016). More precisely, two influential parameters (Scaling and Asymmetry) indicated respectively that participants updated less than predicted by Bayes' rule, and that the rationality was even more reduced (i.e., updates were even smaller) after bad news relative to good news. Particularly the influence of Asymmetry confirms the effect of valence on belief updating.

Moreover, we demonstrated that belief updates can be formalized in a simpler and superior way than suggested by Shah et al. (2016). This alternative formalization based on reinforcement learning framework confirmed the importance of three aspects of belief updating: (i) valence-dependent asymmetry in updates, (ii) the influence of personal knowledge, and (iii) lower updates than predicted by the estimation error. The first aspect is in line with the task performance results and the “optimistic” Bayesian model. The comparison of alternative models showed that learning from estimation errors was indeed asymmetric. Learning rates were greater in response to positive errors (i.e., good news) than negative errors (i.e., bad news), confirming again the influence of valence on belief formation while formally controlling for other trial-wise task parameters. Moreover, this asymmetry in learning rates was strongly related to the asymmetry in participants' updates. Thus, the valence-dependent updating was determined by how much bad news was taken into account during belief formation relative to good news, but not by other features of the belief formation relating to cognitive factors.

Furthermore, model selection revealed that personal knowledge indeed played a significant role during the updating of beliefs about future outcomes. This means that the more participants perceived themselves to be different from average, the less they took the information about the average risk into account. Notably, the winning formalization (“1 – rP” instead of “1 – rP ^*^ W”) implicates that extreme values of personal knowledge will lead either to full consideration of estimation error (when one is equally at risk as the average person; however, there is still an influence of learning rate and valence), or to no consideration at all and thus no updating (when one is maximally different from the average person, independent of learning rate and valence). While this appears trivial at first glance, it does not necessarily represent a reasonable updating in the context of the present task. Even if there is an a priori perceived difference between the estimated base rate (e.g., 20%) and the own risk (e.g., 10%), learning about the actual base rate (e.g., 25%) may be expected to shift the estimate of the own risk by the size of the estimation error (from 10 to 15% due to the difference between the estimated and the actual base rate of 5%). Personal knowledge has long been considered to play an important role in the research area of unrealistic optimism (Shepperd et al., 2002; note that the focus here is on beliefs *per se*, independent of their updating). While personal information was already considered by Shah et al. (2016), however by using different formalizations, they did not explicitly test whether it indeed made a significant contribution to the update dynamics. Providing this proof within a belief update task has important clinical implications. Health promotion and disease prevention management need to take into account that changes in existing beliefs may be affected by perceived personal distance to the referred populations (Shepperd et al., 2002).

The third aspect of belief updating—the updates lower than predicted by the error—may relate to varying degrees of precision, or uncertainty, of beliefs and new information. For instance, generally reduced updating could result from low trust of presented base rates relative to high certainty of initial beliefs about base rates and own risks. Indeed, while none of the included participants doubted the general creditability of the presented (manipulated) base rates, 60% reported that some of them appeared odd. Thus, assessment of participants' certainty about their probability estimates, and their trust in the presented base rates could help to optimize further computational models. Moreover, using expressions of certainty as dependent variables may improve the understanding of motivational influences on expectations (Krizan and Windschitl, 2007). For instance, higher certainty about subjective probability estimates after desired updates than after unfavorable updates could provide a further support for the influence of valence on predictions.

Related to this, the superiority of the alternative model does not suggest that reinforcement learning is a better mechanistic explanation for belief updating than the Bayesian theorem. The two frameworks share central assumptions and differ mainly with respect to the consideration of precision (included in Bayesian models, but not in reinforcement learning ones). Therefore, the model based on reinforcement learning might be justified merely by limited data obtained with the current design, which does not include measures of subjective belief precision. Another reason that might explain the failure of the Bayesian model (beyond the valence effect) relates to the way belief precision is formally implemented. Building on the work of Shah et al. (2016), we captured beliefs probabilities as simple point estimates. Mathematically, this corresponds to representing life events as following a Bernouilli distribution. This assumption, the simplest model for representing the probability of a binary outcome, enforces a specific relationship between the believed expected probability of an event and the precision of that belief. More precisely, beliefs about extreme probabilities, such as 2 or 97% risks, will always be associated with a high degree of certainty, whereas a belief of a 50% risk will always be the most uncertain (formally, if an event is believed to happen with a probability *p*, the uncertainty about this event is *p(1-p)*). However, this assumption is unlikely to hold in real life: one can be quite certain that a coin toss has a 50% chance to land tail, or be very uncertain about the probability of receiving a pine cone on their head, although knowing this event should be rater rare^3^. A way to overcome this problem is to model beliefs utilizing a distribution having a parametric variance, e.g., a Beta distribution. Practically, this means that the experimenter would have to ask for each event how confident the subject is about the estimates (eBR, E1, E2, and also BR). In a Bayesian setting, the different estimates will then be weighted according to their respective precision when entering the update rule. Such models could be useful to explain the trial-by-trial variations in update size (corresponding to varying levels of certainty about estimated base rates) or why subjects do not fully correct their estimation error (because they don't completely trust the numbers given by the experimenter). However, this added complexity is unlikely to explain the optimism bias we observed in our data. As all events and errors are randomized within and between subjects, the effects of belief precision should cancel each other out across the conditions.

While the psychological and neurobiological evidence for differential processing of desirable and undesirable new information and resulting belief updates (Yacubian et al., 2006; Sharot et al., 2011; Moutsiana et al., 2015; Palminteri et al., 2015, 2016; Kuzmanovic et al., 2016a; Lefebvre et al., 2017) emphasizes the need to find a principled computational explanation of the valence-induced bias *per se*, this endeavor is more difficult. Here, we adopted a bottom-up approach to try to single out the mechanistic components on which a more general theory of belief updating could build upon. Another approach is to start from conceptual axioms to derive a normative theory of valence-dependent cognition. Some authors have already proposed promising reflections in this direction by attributing emotional value to beliefs (Mobius et al., 2011), or by integrating agency into belief updating (active inference, Friston et al., 2013). However, those theories rely on (sometimes implicit) theoretical assumptions and make intricate predictions that need further experimental consideration.

## Author contributions

BK developed the study concept and the study design, and performed the testing and data collection. BK and LR performed the data analysis, the interpretation and wrote the manuscript. Both authors agree to be accountable for the content of the work.

### Conflict of interest statement

The authors declare that the research was conducted in the absence of any commercial or financial relationships that could be construed as a potential conflict of interest.

## Footnotes

^1^In addition to the valence-dependent bias, i.e., the tendency to draw the conclusion that a choice option is rewarding, Palminteri and colleagues also demonstrated a confirmatory bias, i.e., the tendency to draw a conclusion that one made the correct choice. Thus, confirming a preexisting belief seems to be similarly desirable as obtaining an external reward.

^2^Difference measure (predicted belief change—observed belief change, values closer to zero represent more normative belief updating) was valence-dependent only in two of four experiments: experiment 2 [good news: *M* = 1.72, *SD* = 1.95, bad news: *M* = 7.62, *SD* = 5.96, *t*_(16)_ = 4.37, *p* < 0.001, *d* = 1.12] and experiment 3B [descriptive statistics not reported, but *t*_(111)_ = 1.95, *p* = 0.054, only a trend-level]; descriptive statistics for experiment 3A and 4 not reported.

^3^http://www.wired.com/2010/11/what-are-the-chances-of-getting-hit-by-a-falling-pine-cone/
