# Supplementary material for: Valence-Dependent Belief Updating: Computational Validation
Source: Front Psychol. 2017 Jun 29;8:1087. doi: 10.3389/fpsyg.2017.01087 (PMC5489622; doi:10.3389/fpsyg.2017.01087)
Supplement: Supplementary file 1 [file Table1.docx]

Supplementary Material

Valence-Dependent Belief Updating: Computational Validation

Bojana Kuzmanovic*, Lionel Rigoux

*** Correspondence:** Bojana Kuzmanovic: bojana.kuzmanovic@sf.mpg.de

# Complete List of Stimulus Events

| **Original stimuli (German)** | **English translation** |
| --- | --- |
| altersbedingte Blindheit | age related blindness |
| altersbedingte Schwerhörigkeit | age related deafness |
| Altersdemenz | dementia |
| Angststörung | anxiety disorder |
| Arbeitslosigkeit | unemployment |
| Arbeitsunfall | work accident |
| Armut | poverty |
| Arthritis (Gelenkentzündung) | arthritis (joint inflammation) |
| Arthrose (Gelenkverschleiß) | wear of the joint |
| Asthma bronchiale | bronchial asthma |
| Autodiebstahl | car theft |
| Autoimmunerkrankung | autoimmune disease |
| Bauchspeicheldrüsenkrebs | pancreas cancer |
| Belastung durch Inflation | inflation |
| Belastung durch Luftverschmutzung | exposure to air pollution |
| Betrug beim Internetkauf | fraud when when purchasing online |
| Betrug mittels Datendiebstahl | computer fraud by data theft |
| Betrug mittels Kreditkartendiebstahl | card fraud |
| Betrug mittels Lastschriftverfahren | fraud by automatic debit transfer |
| Blinddarmentzündung | infection of the appendix |
| Blutgerinnsel | blood clot |
| Bluthochdruck | chronic high blood pressure |
| Burn-Out | burnout |
| chronische Bronchitis | chronic bronchitis |
| Computerabsturz mit Datenverlust | computer crash with loss of data |
| Darmkrebs | bowel cancer |
| Darmspiegelung | colonoscopy |
| Depression | depression |
| Drogen-/Medikamentenabhängigkeit | drug addiction |
| Erkrankung des Rückenmarks | disease of the spinal cord |
| Fahrraddiebstahl | bicycle theft |
| Flöhe/Läuse bekommen | having fleas/lice |
| Flug verpassen | miss a flight |
| gefeuert werden | be fired |
| Geschwür | ulcer |
| Glutenunverträglichkeit | intolerance to gluten |
| grauer Star | cataract |
| Harninkontinenz (unfreiwilliger Harnverlust) | urinary incontinence |
| Hepatitis A oder B | hepatitis A or B |
| Herzinfarkt | heart attack |
| Heuschnupfen | hay fever |
| Knochenbruch | bone fracture |
| koronare Herzerkrankung (verengte Herzkranzgefäße) | arteries hardening (narrowing of blood vessels) |
| körperliche Gewalt | victim of violence |
| Krampfadern | varicose veins |
| künstliches Gelenk | artificial joint |
| lärminduzierte Minderung der Hörfähigkeit | noise-induced hearing-loss |
| Leistenbruch | inguinal hernia |
| Lungenkrebs | lung cancer |
| Magenkrebs | gastric cancer |
| medizinischer Behandlungsfehler | medical malpractice |
| mehr als 30000 EUR Schulden | debt of more than 30000 EUR |
| Migräne | migraine |
| Nierensteine | kidney stones |
| Osteoporose (Knochenbrüchigkeit) | osteoporosis (reduced bone density) |
| Parkinson-Erkrankung | Parkinson’s disease |
| Pflegebedürftig sein | dependent on nursing care |
| psychische Gewalt (z.B. Mobbing) | victim of psychological harassment |
| Reizdarmsyndrom | irritable bowel syndrome |
| riskanter Alkoholkonsum | hazardous alcohol consumption |
| Rückenschmerzen | back pain |
| Schädlingsbefall in der Wohnung | pest infestation |
| Schilddrüsenerkrankungen | thyroid disorders |
| Schlafstörungen | insomnia |
| Schlaganfall | having a stroke |
| Schuppenflechte | psoriasis |
| schwere Zahnprobleme | serious dental problems |
| sexuelle Dysfunktion | sexual dysfunction |
| Straßendiebstahl | mugging |
| Tinnitus | tinnitus |
| Typ-2-Diabetes (tritt erst im Erwachsenenalter auf) | diabetes type 2 (onset in adulthood) |
| Übergewicht | overweight |
| Unfruchtbarkeit | infertility |
| unzureichende Rentenversorgung im Alter | insufficient pension provision |
| Venenerkrankungen | venous diseases |
| Verkehrsunfall | traffic accident |
| Verletzung durch einen Unfall | injury caused by an accident |
| vom Partner/Partnerin betrogen werden | being cheated by your partner |
| Wohnungseinbruchsdiebstahl | domestic burglary |
| Zeuge eines traumatisierenden Unfalls sein | witness a traumatizing accident |
| **Events used during the training session** | |
| Fensterscheibenbruch | broken window |
| Haarausfall | hair loss |
| Scheidung | divorce |
| Urlaubsabbruch | interruption of a vacation |
| Wasserrohrbruch | burst water-pipe |
| Zahnfleischprobleme | gum problems |

# Manipulation of Presented Base Rates

As described in the manuscript, the base rates presented in the belief update experiment were systematically manipulated, capped between 1 and 90% (the upper bound was lower than 100% in order to prevent conspicuously high base rates). First, the values [5, 7, 9, 11, 13, 16, 18, 20, 22, 24] were randomly assigned to the first 10 GOOD trials (BR < eBR), and the values [6, 8, 10, 12, 14, 15, 17, 19, 21, 23] were randomly assigned to the first 10 BAD trials (BR > eBR). In GOOD trials, the values were subtracted from respective eBR, and in BAD trials, the values were added to eBR.

The two following deviations from this simple algorithm were possible. First, in GOOD trials, the eBR could be smaller than the randomly assigned value (e.g., eBR = 10%, but value = 20). In these cases, the array of remaining values was scanned in order to find a value that is smaller than eBR (e.g., 5). If the search was successful, the two values were replaced (the non-used value 20 was scheduled instead of the substitute value 5 to be used in a subsequent trial). If this was not successful (because the eBR was too small, or there was no appropriate not yet used substitute value in the array), BR was capped to 1%, resulting in a smaller EE than originally scheduled (e.g., if eBR = 10%, and value = 20, then BR = 1%, and EE = 9% instead of 20%).

Second, in BAD trials, the sum of eBR and the scheduled value could be greater than 90. Again, in these cases, the array of remaining values was scanned in order to find a substitute value that is smaller, so that the sum of eBR and the substitute value is <= 90%. If this was not successful, the BR was corrected to a value randomly selected from a range between 85 and 90%, again resulting in a different EE than originally scheduled (e.g., if eBR = 85%, and value = 10, then BR = 87%, and EE = 2% instead of 10%). The random selection from a range of values sought to prevent overly frequent presentations of a base rate of exactly 90%.

Together, these deviations from the original algorithm (when the too large EE value could not be replaced by an appropriate substitute and BR had to be capped) rendered the exact EE sizes between GOOD and BAD trials less comparable. In order to adjust for these differences, we mirrored the realized (not the scheduled) values for the next 20 trials: the realized GOOD values were scheduled for the next 10 BAD trials, and the realized BAD values were scheduled for the next 10 GOOD trials. This procedure was repeated four times during the experiment to accomplish the total of 80 trials.

In the special case of EE of 0 (e.g. if eBR = 1% in GOOD trials, it was not possible to generate an EE, as there is no smaller value than 1), the trial was excluded from analyses (< 1%). In these cases, the scheduled value was kept as the ‘realized’ value for the mirror procedure.

Note that the manipulation procedure with the build-in corrections (search for a substitute EE value, and mirroring of realized EE values across conditions) represents an improvement relative to the previous studies (Kuzmanovic et al., 2015, 2016), and results in a more balanced EE sizes across conditions.
